# Mesenchymal Stromal (Stem) Cell Therapy Fails to Improve Outcomes in Experimental Severe Influenza

**DOI:** 10.1371/journal.pone.0071761

**Published:** 2013-08-15

**Authors:** Ilyse Darwish, David Banner, Samira Mubareka, Hani Kim, Rickvinder Besla, David J. Kelvin, Kevin C. Kain, W. Conrad Liles

**Affiliations:** 1 Faculty of Medicine, Institute of Medical Science, University of Toronto, Toronto, Ontario, Canada; 2 Sandra A. Rotman Laboratories, Sandra Rotman Centre for Global Health, University Health Network-Toronto General Hospital, Toronto, Ontario, Canada; 3 Division of Experimental Therapeutics, Toronto General Research Institute, University Health Network, Toronto, Ontario, Canada; 4 Department of Microbiology, Sunnybrook Health Sciences Centre and Research Institute and Department of Laboratory Medicine and Pathobiology, University of Toronto, Toronto, Ontario, Canada; 5 Division of Infectious Diseases, Department of Medicine, University of Toronto, Toronto, Ontario, Canada; 6 Department of Medicine, University of Washington, Seattle, Washington, United States; University of Pittsburgh, United States of America

## Abstract

**Rationale:**

Severe influenza remains a major public health threat and is responsible for thousands of deaths annually. Increasing antiviral resistance and limited effectiveness of current therapies highlight the need for new approaches to influenza treatment. Extensive pre-clinical data have shown that mesenchymal stromal (stem) cell (MSC) therapy can induce anti-inflammatory effects and enhance repair of the injured lung. We hypothesized that MSC therapy would improve survival, dampen lung inflammation and decrease acute lung injury (ALI) in a murine model of severe influenza**.**

**Methods:**

C57Bl/6 mice were infected with influenza A/PuertoRico/8/34 (mouse-adapted H1N1) or influenza A/Mexico/4108/2009 (swine-origin pandemic H1N1) and administered human or mouse MSCs via the tail vein, either pre- or post- infection. MSC efficacy was evaluated as both an independent and adjunctive treatment strategy in combination with the antiviral agent, oseltamivir. Weight loss and survival were monitored. Inflammatory cells, cytokine/chemokines (IFN-γ, CXCL10, CCL2 and CCL5) and markers of ALI (total protein and IgM), were measured in bronchoalveolar lavage fluid and lung parenchyma.

**Results:**

Administration of murine MSCs or human MSCs in a prophylactic or therapeutic regimen failed to improve survival, decrease pulmonary inflammation/inflammatory cell counts or prevent ALI in influenza virus-infected mice. MSCs administered in combination with oseltamivir also failed to improve outcomes.

**Conclusions:**

Despite similarities in the clinical presentation and pathobiology of ALI and severe influenza, our findings suggest that MSC therapy may not be effective for prevention and/or treatment of acute severe influenza.

## Introduction

While the vast majority of influenza A virus infections resolve without complications, approximately 3–5 million affected individuals worldwide develop severe and potentially fatal disease annually [1]. Severe influenza can activate deleterious innate immune responses and cause acute lung injury (ALI)/acute respiratory distress syndrome (ARDS), which directly contribute to influenza-associated morbidity and mortality [2]–[14]. ALI/ARDS is characterized by increased permeability of the microvascular endothelium and disruption of the alveolar-capillary membrane barrier, leading to pulmonary edema accompanied by neutrophil, macrophage, and erythrocyte infiltration [15], [16].

The two classes of FDA approved drugs for the prevention and treatment of influenza - the adamantanes and the neuraminidase inhibitors - have several limitations in clinical practice. Frequent mutations and gene reassortments between influenza A viruses have resulted in decreased efficacy of antiviral therapy. For example, the development of drug resistance to adamantanes has rendered this class of drugs ineffective [17]. A significant increase in seasonal influenza A (H1N1) virus mutations conferring resistance to the neuraminidase inhibitor oseltamivir has also been observed [18]. Efficacy of antiviral therapy also depends on the timing of administration [19]–[23]. Initiation of oseltamivir treatment within the first 48–72 hours after the onset of influenza symptoms reduces mortality [20], [21]. On the other hand, oseltamivir treatment initiated beyond the first 48–72 hours after the onset of influenza symptoms has limited clinical impact [20], [21]. In addition, neuraminidase inhibitors have been reported to be relatively ineffective in H5N1 avian influenza virus infection [24]. Development of novel adjunctive treatment strategies to complement antiviral therapy might improve clinical outcome in severe influenza.

Mesenchymal stromal (stem) cells (MSCs) represent a potential immunomodulatory strategy for treatment of ALI [25]–[32]. MSCs are a heterogenous subset of non-hematopoeitic pluripotent stromal cells with multilineage potential that can be isolated from embryonic tissue, adipose tissue, liver, muscle and dental pulp; however, adult bone marrow remains the most common source of MSCs for pre-clinical and clinical studies [33], [34]. Initial clinical interest in MSCs focused on their ability to differentiate into injured cell types as a means to improve outcome in pre-clinical models of disease. More recent studies have demonstrated that MSCs can reduce injury without engraftment in murine models of endotoxin, bleomycin or *Escherichia coli*-induced ALI and sepsis [25]–[28], [35]. Potential mechanisms underlying the protective role of MSCs in ALI include immunosuppression, enhanced repair of the injured lung and enhanced bacterial clearance [25]–[28], [35]. These effects have been shown to be mediated by direct MSC-cell contact with host cells, MSC secretion of various paracrine mediators of inflammation (e.g., TNF-stimulated gene 6 protein (TSG-6) and prostaglandin E2 (PGE_2_)) and MSC upregulation of antimicrobial factors [25]–[32]. A recent transcriptomic analysis demonstrated that MSC administration modulated a broad range of biological networks in the lung that protect from tissue injury [36].

These pre-clinical data suggest the possibility that MSCs could be used to improve clinical outcomes in severe influenza. In this study, we investigated whether administration of MSCs in a model of experimental severe influenza, either independently or as an adjunctive treatment strategy, could improve clinical outcome by decreasing influenza-induced inflammation and ALI.

## Materials and Methods

### Murine Influenza Model

Male C57Bl/6 mice, 7–10 weeks old, were obtained from Jackson Laboratories and maintained under pathogen-free conditions with a 12-hour light cycle. Animal use protocols were reviewed and approved by the University Health Network Ontario Cancer Institute Animal Care Committee, and all experiments were conducted in accordance with institutional guidelines in an animal biosafety level 2 facility. On day 0, under light isofluorane anesthesia, experimental mice were infected by nasal instillation with 425 50% egg infectious doses (EID_50_) (60–80% lethal dose) or 150 EID_50_ (non-lethal dose) influenza A/PuertoRico/8 virus (A/PR/8; mouse-adapted H1N1) (ATCC; Lot#3628278) or 1000 EID_50_ influenza A/Mexico/4108/2009 (A/Mex/4108; 2009 swine-origin pandemic H1N1) (Centers for Disease Control; Lot#200912192). Weight was recorded daily for a maximum of eleven days and mice were sacrificed either on day 7 or when euthanasia criterion was met (≤80% of day 0 weight).

### Influenza Virus Titration

On day 6 post-infection, C57Bl/6 mice were euthanized and lungs were harvested. Lungs were weighed and homogenized in 1 ml PBS for 30 sec. Lung homogenates were spun at 10,000 *g* for 10 min and the supernatant was stored at −80°C. Influenza A/PR/8 viral yield was quantified by plaque assay in Madin-Darby canine kidney (MDCK) cells (ATCC). 1×10^6^ MDCK cells/well were plated in 6-well plates. 12–24 hours later, medium was removed and 10-fold dilutions of lung homogenate in 500 µL serum-free Eagle’s minimum essential media (MEM) were added (in duplicate) to MDCK cells and incubated for 1 hour at 37°C with 5% CO_2_. Cells were then overlaid with 2 mL of 1×Eagle’s MEM containing 0.6% agarose, antibiotics, sodium bicarbonate and 8 µl trypsin. Cells were incubated for 42–72 hours then fixed with Carnoy’s fixative (3∶1, methanol:glacial acetic acid) for 30 min. The agarose overlay was then removed and cells were stained with 0.1% crystal violet in 20% ethanol to visualize plaques. Viral load was expressed as plaque forming units per gram of lung tissue (PFU/g).

### Mesenchymal Stromal (Stem) Cells (MSCs)

Frozen vials of syngeneic bone marrow-derived murine MSCs (mMSCs) and allogeneic human MSCs (hMSCs) were obtained from Dr. Darwin Prockop, Texas A&M Health Science Center College of Medicine Institute for Regenerative Medicine at Scott & White (Temple, TX, USA), under the auspices of a National Institutes of Health/National Centre for Research Resources (NIH/NCRR) grant (# P40RR017447). All MSCs were reported by the Centre as meeting MSC defining criteria proposed by the International Society for Cellular Therapy (ISCT) [37] and have previously been effective in reducing LPS- and sepsis- induced ALI in murine models [26], [35], [38]. Differentiation of MSCs was evaluated using the Mesenchymal Stem Cell Functional Identification Kit (R&D Systems), as per manufacturer’s instructions. To examine MSC surface antigen expression, hMSCs (P3) were labeled with anti-human antibodies CD73, CD90, CD105, CD11b, CD19, CD34, CD45, HLA-DR (as well as isotype/compensation controls) (Human MSC Analysis Kit; BD Biosciences). All cells were analyzed by flow cytometry. mMSCs (P9) and hMSCs (P3) retained the ability to differentiate into three cellular lineages including adipocytes, osteocytes and chondrocytes, under standard *in vitro* differentiating conditions (Figure S1A,B). hMSCs (P3) were >99% positive for stem cell surface antigens CD73, CD90, and CD105 and <2% positive for hematopoietic cell markers CD11b, CD19, CD34, CD45, HLA-DR, thereby fulfilling ISCT MSC defining criteria [37] (Figure S1C).

mMSCs (isolated from male C57Bl/6 mice) were thawed and plated for 24 hours in α-MEM, without ribonucleosides or deoxyribonucleosides, and supplemented with antibiotics, 10% fetal bovine serum (FBS) (Atlanta Biologicals) and 10% horse serum. hMSCs (isolated from a 24 year old male donor) were thawed and plated for 24 hours in α-MEM, without ribonucleosides or deoxyribonucleosides, supplemented with 2 mM L-glutamine, antibiotics and 16.5% FBS. After 24 hrs, mMSC or hMSCs were trypsinized and re-plated at 60 cells/cm^2^. mMSCs/hMSCs were incubated for each subsequent passage until cells were ∼70% confluent. P6–P9 mMSCs or P3 hMSCs were re-suspended in PBS and 2.5×10^5^ cells, 5×10^5^ cells or PBS alone was administered, via the tail vein, into experimental mice on day −2, 0, 2 or 5 post-infection. Injections were performed using 26.5 gauge needles and a typical mouse restrainer.

### Oseltamivir

Oseltamivir phosphate from capsules (Tamiflu) (Roche) was dissolved in ddH_2_O and experimental mice were administered 2.5 mg/kg by oral gavage, once daily, beginning 2 days post-infection for a maximum of 5 days. Control mice were administered ddH_2_O via oral gavage.

### Bronchoalveolar Lavage (BAL) Fluid Analysis

C57Bl/6 mice were euthanized and BAL fluid from both lungs was obtained by three consecutive instillations and aspirations of 500 µl sterile PBS. Aliquots were spun at 800 *g*, 4°C, for 5 min. Supernatant from the first lavage was removed and stored at −80°C for further analysis. Cells from all aliquots per mouse were combined and counted using a hemocytometer. Differential inflammatory cell counts were determined by cytocentrifugation and modified Wright-Giemsa staining. BAL fluid concentration of CCL2, CXCL10, CCL5 and IFN-γ was measured by sandwich ELISA (DuoSet, R&D Systems for CCL2, CXCL10, CCL5; eBioscience for IFN-γ) according to the manufacturer’s instructions. BAL fluid total protein concentration was measured using a BCA protein assay (Sigma-Aldrich) and BAL fluid IgM concentration was measured by sandwich ELISA (Bethyl Laboratories) according to the manufacturer’s instructions.

### Lung Homogenate Analysis

C57Bl/6 mice were euthanized and lungs were harvested. Lungs were weighed and homogenized in 2 ml PBS/g lung tissue for 30 sec. Lung homogenates were spun at 10,000 *g,* 4°C, for 10 min and the supernatant was stored at −80°C. Cytokine and chemokine concentrations were measured as described above.

#### Lung histology

C57Bl/6 mice were euthanized on day 7 post-infection and perfused with 10 ml PBS. Formalin was injected into the trachea until the lungs were inflated (approximately 2 ml). Lungs were excised and immersed in formalin for 24 h followed by transfer to 70% ethanol. Processed and paraffin wax-embedded sections were stained with hematoxylin and eosin (H&E).

### Statistical Analysis

Analysis was performed using GraphPad Prism v4 software. Kaplan-Meier survival curves were compared using the logrank test. Differences between groups were assessed by one- or two-way analysis of variance (ANOVA) with Bonferroni post-tests. P<0.05 was considered statistically significant. All normally distributed data (weight) are expressed as mean ± standard deviation (SD). All non-normally distributed data (protein level and viral load) are expressed as median ± interquartile range (IQR).

## Results

### Neither Prophylactic Nor Therapeutic Administration of mMSCs Affected Weight Loss or Improved Survival in Two Models of Experimental Severe Influenza

C57Bl/6 mice were infected intranasally with 425 EID_50_ influenza A/PR/8 (mouse-adapted H1N1) or 1000 EID_50_ influenza A/Mex/4108 (2009 swine-origin pandemic H1N1). mMSCs (2.5×10^5^ cells) were administered to influenza virus-infected mice via the tail vein, either prophylactically (4 hours prior to infection and 2 days post-infection) or therapeutically (day 2 or day 5 post-infection).

**Figure 1:**
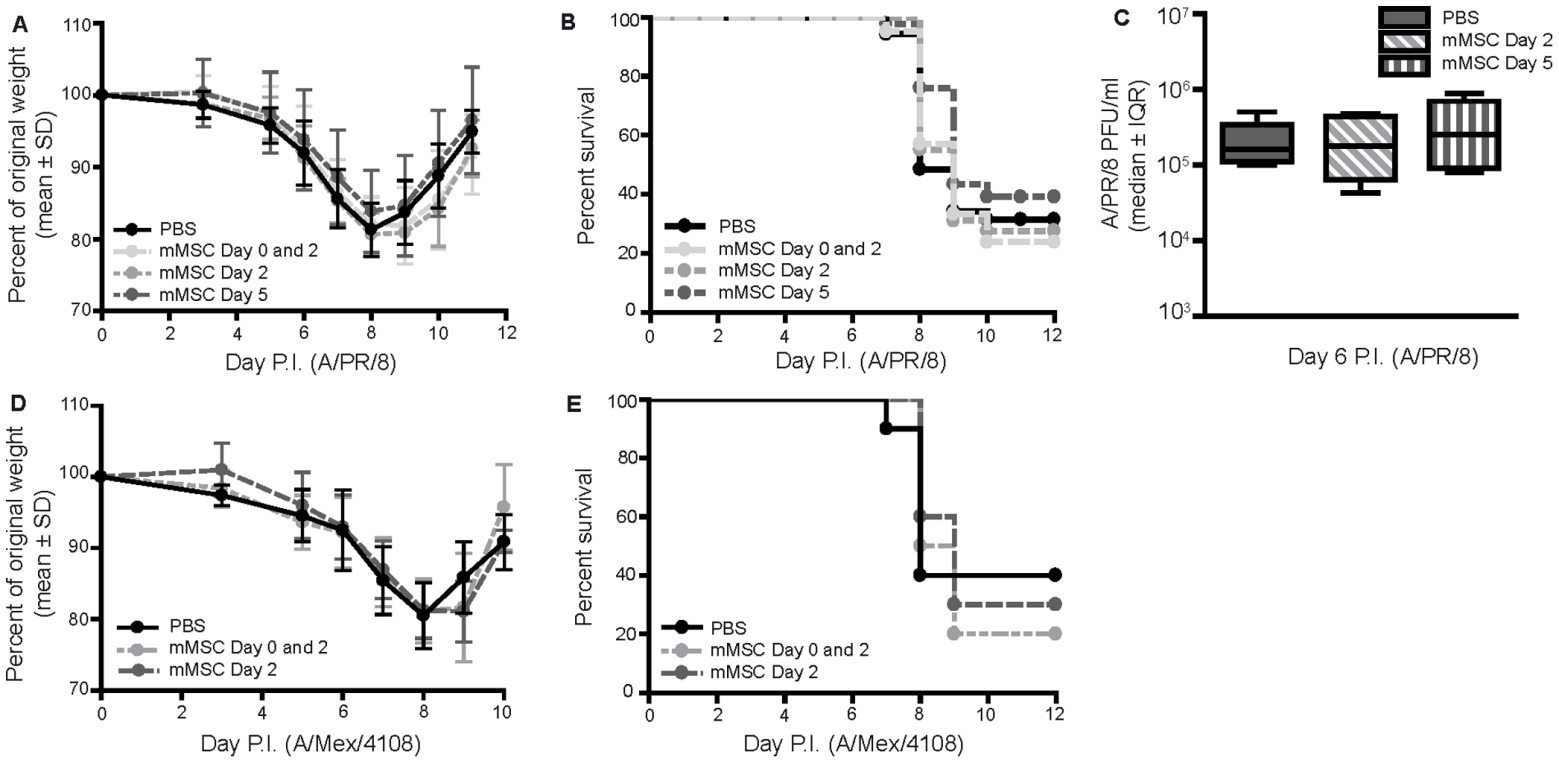
Neither prophylactic nor therapeutic administration of mMSCs affected weight loss or improved survival in two models of experimental severe influenza. 7–10 week-old male C57Bl/6 mice were (**A,B**) infected with 425 EID_50_ influenza A/PR/8 virus and administered 2.5×10^5^ mMSCs (passage 6–9), intravenously, either prophylactically (4 hours prior to infection and day 2 post-infection (P.I.)) or therapeutically (day 2 or day 5 P.I.). No significant differences in weight loss kinetics (Two-way ANOVA, n = 21–46/group, 3 pooled experiments) or survival (logrank test, n = 21–46/group, 3 pooled experiments) were observed. Error bars represent standard deviation. (**C**) Lungs were harvested on day 6 P.I. and viral load was quantified via plaque assay in MDCK cells. No significant differences were observed (one-way ANOVA, n = 5/group). Error bars represent interquartile range (IQR). PFU = plaque forming units. (**D,E**) Mice were infected with 1000 EID_50_ influenza A/Mex/4108 and administered mMSCs prophylactically (4 hours prior to infection and day 2 P.I.) or therapeutically (day 2 P.I.). No significant differences in weight loss kinetics (two-way ANOVA, n = 10/group) or survival (logrank test, n = 10/group) were observed. Error bars represent standard deviation.

Influenza A/PR/8 or A/Mex/4108 infected mice, administered mMSCs prophylactically or therapeutically, experienced similar weight loss kinetics and consequent survival kinetics compared to infected control mice administered PBS (Figure 1A,B,D,E). No difference in lung viral titer was observed for influenza A/PR/8 infected mice administered mMSC on day 2 or day 5 post-infection compared to infected control mice (Figure 1C).

### Therapeutic Administration of mMSCs Failed to Decrease Pulmonary Inflammation and Inflammatory Cell Counts or Prevent ALI in Experimental Severe Influenza

**Figure 2:**
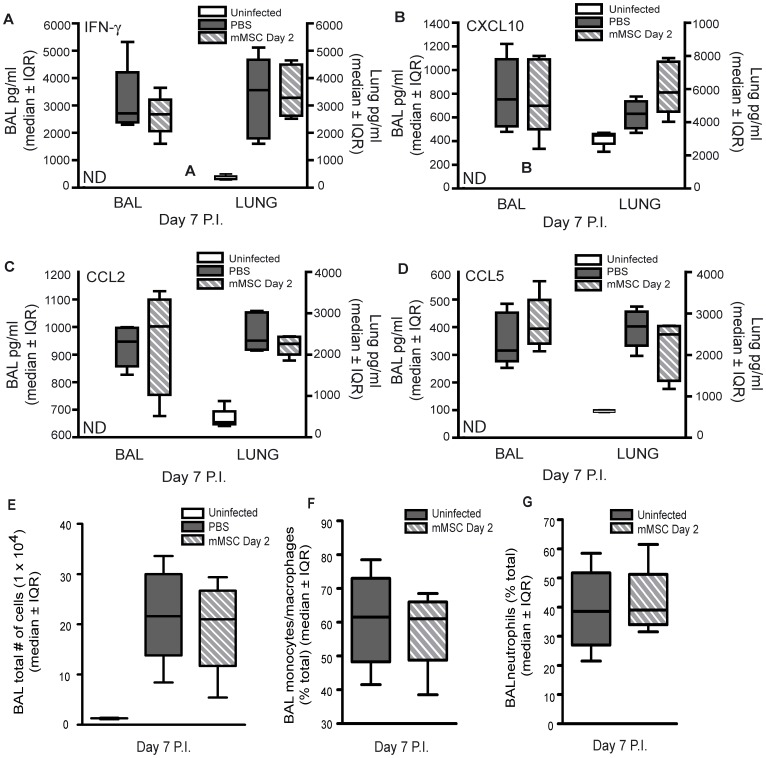
Therapeutic administration of mMSCs failed to decrease pulmonary inflammation or modify BAL inflammatory cell counts in experimental severe influenza. 7–10 week-old male C57Bl/6 mice infected with 425 EID_50_ influenza A/PR/8 virus and administered 2.5×10^5^ mMSCs (passage 6–9) or PBS on day 2 P.I. were sacrificed on day 7 P.I. BAL fluid and lungs were harvested. (**A–D**) No significant difference in BAL fluid and lung homogenate cytokines and chemokines (IFN-γ, CXCL10, CCL2 or CCL5) was observed for mice administered mMSCs compared to infected control mice administered PBS (One-way ANOVA, n = 4–6/group, representative of 2 independent experiments). (**E**) The total number of BAL inflammatory cells was similar for mMSC-treated mice and infected control mice administered PBS (one-way ANOVA, n = 6/group). There was no significant difference in the total percentage of BAL (**F**) monocytes/macrophages and (**G**) neutrophils between mice administered mMCS and infected control mice administered PBS (one-way ANOVA, n = 6/group). Error bars represent interquartile range (IQR). ND = nondetectable.

Because MSCs have been reported to have potent effects on pulmonary inflammation and ALI in a number of pre-clinical models [25]–[32], we sought to determine whether mMSC administration decreased pulmonary inflammation or prevented ALI in experimental severe influenza. C57Bl/6 mice were infected with 425 EID_50_ influenza A/PR/8 and administered 2.5×10^5^ mMSCs or PBS via the tail vein on day 2 post-infection. Uninfected control mice were administered PBS intranasally. All experimental mice were sacrificed on day 7 post-infection. BAL fluid and lung homogenate concentrations of cytokines/chemokines (IFN-γ, CXCL10, CCL2 and CCL5) were measured (Figure 2A–D). Cytokine/chemokine levels were below the limit of detection in BAL fluid of uninfected control mice and significantly lower in lung homogenate of uninfected control mice compared to infected mice. All BAL fluid and lung homogenate cytokine/chemokine levels were elevated on day 7 post-infection. Cytokine/chemokine levels in BAL fluid and lung homogenate of influenza virus-infected mice were unaffected by mMSC administration (Figure 2A–D). BAL inflammatory cell counts, including monocytes/macrophages and neutrophils, were also unaffected by mMSC administration (Figure 2E–G).

**Figure 3:**
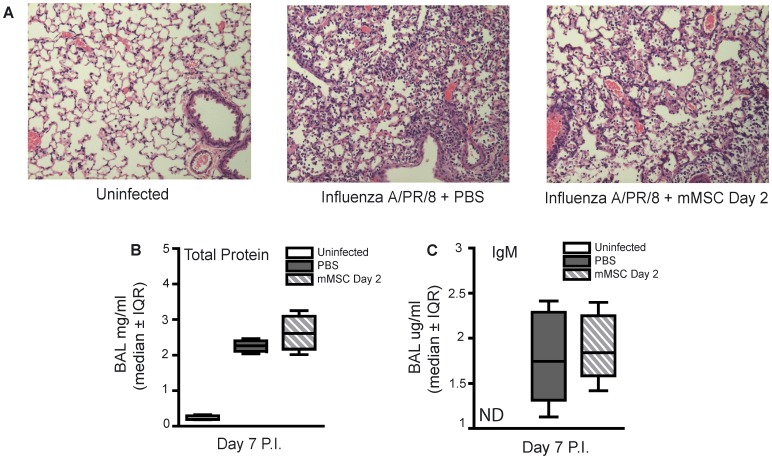
Therapeutic administration of mMSCs failed to modify pulmonary inflammation or alter acute lung injury in experimental severe influenza. Eight week old male C57Bl/6 mice were infected with 425 EID_50_ influenza A/PR/8 virus and administered 2.5×10^5^ mMSCs (passage 6), via the tail vein, on day 2 P.I. Mice were sacrificed on day 7 P.I. (**A**) Representative images of hematoxylin and eosin stained lung sections demonstrate similar cellularity and lung injury in mMSC treated mice compared to infected controls. (**B,C**) No significant difference in BAL fluid markers of ALI (total protein or IgM) was observed between mice administered mMSCs and infected control mice administered PBS (one-way ANOVA, n = 6/group, representative of 2 independent experiments). Error bars represent interquartile range (IQR). ND = nondetectable.

On day 7 post-infection, similar inflammatory cell infiltrates were observed in the lungs of mMSC-treated mice compared to infected controls, as shown by histology (Figure 3A). Total protein and IgM were significantly lower or below the level of detection in BAL fluid of uninfected control mice compared to infected mice, respectively (Figure 3B,C). However, no difference in total protein or IgM was observed for mice administered mMSCs compared to control mice administered PBS (Figure 3B,C).

### Neither Prophylactic Nor Therapeutic Administration of hMSCs Affected Weight Loss or Improved Survival in either Experimental Severe or Sub-lethal Influenza

Most experimental evidence to date regarding the use of MSC treatment for ALI has been derived from studies that employed mMSCs [25], [26], [29], [31]. However, it is critical that the efficacy and mechanism of hMSCs be studied in depth before proceeding to clinical trials, so that the human physiological response could be accurately predicted [38], [39], [40]. Evidence also suggests that the mechanism by which hMSCs and mMSCs exert their effects varies in some models [41]; however, hMSC therapy was as effective as mMSC therapy in murine models (eg. LPS-induced ALI) [26], [31], [38]. Therefore, we also evaluated the use of hMSC treatment in experimental severe influenza.

**Figure 4:**
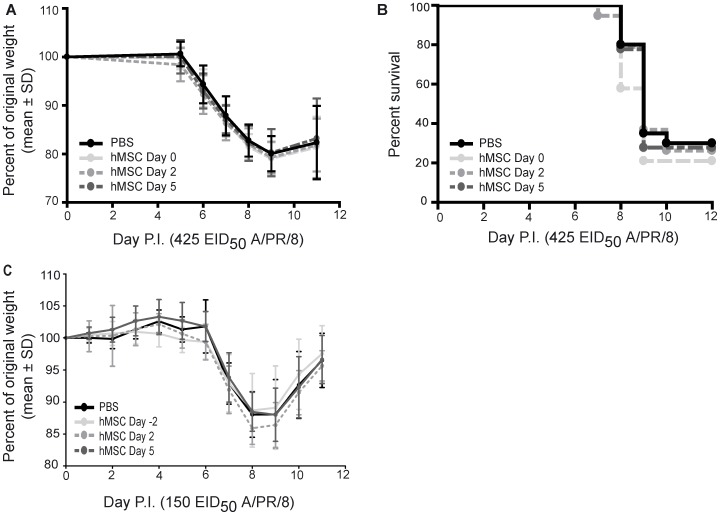
Neither prophylactic nor therapeutic administration of hMSCs altered weight loss or improved survival in either experimental severe or non-lethal influenza. Eight week-old male C57Bl/6 mice were infected with 425 EID_50_ (lethal model) or 150 EID_50_ influenza A/PR/8 virus (sub-lethal model) and administered 2.5×10^5^ hMSCs (passage 3) or PBS, via the tail vein, either prophylactically (2 days or 4 hours (day 0) prior to infection) or therapeutically (day 2 or day 5 P.I.). Weight was recorded daily. No significant differences in (**A**) weight loss kinetics (two-way ANOVA, n = 18–20/group, 2 pooled experiments) or (**B)** survival (logrank test, n = 18–20/group, 2 pooled experiments) were observed in the lethal influenza model. (**C**) No significant differences in weight loss kinetics were observed in the sub-lethal influenza model (two-way ANOVA, n = 11/group). Error bars represent standard deviation.

C57Bl/6 mice were infected with 425 EID_50_ (lethal dose) or 150 EID_50_ (non-lethal dose) influenza A/PR/8, and 2.5×10^5^ hMSCs were administered via the tail vein, either prophylactically (2 days or 4 hours prior to infection; day −2 and day 0, respectively), or therapeutically (day 2 or day 5 post-infection). There was no difference in influenza A-induced weight loss (lethal and non-lethal model) or survival between mice administered hMSCs and control mice administered PBS (Figure 4).

### Therapeutic Administration of hMSCs Failed to Decrease Pulmonary Inflammation and Inflammatory Cell Counts or Prevent ALI in Experimental Severe Influenza

**Figure 5:**
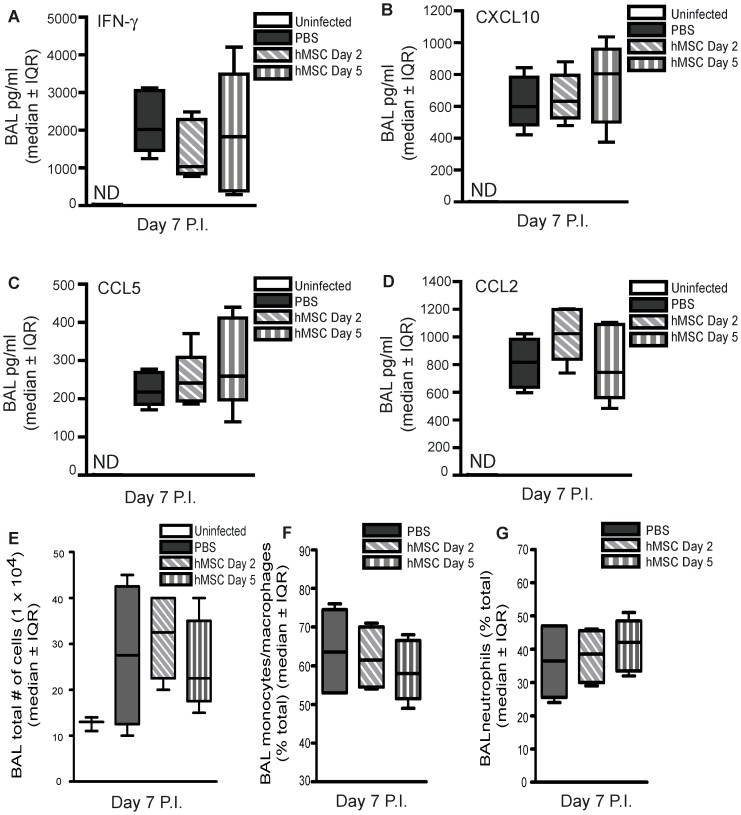
Therapeutic administration of hMSCs failed to decrease pulmonary inflammation or modify BAL inflammatory cell counts in experimental severe influenza. Eight week-old male C57Bl/6 mice infected with 425 EID_50_ influenza A/PR/8 virus and administered 2.5×10^5^ hMSCs (passage 3) on day 2 or day 5 P.I. were sacrificed on day 7 P.I. BAL was performed. (**A–D**) No significant difference in BAL fluid cytokine and chemokines (IFN-γ, CXCL10, CCL2 or CCL5) was observed between mice administered hMSCs on day 2 or day 5 P.I. and infected control mice administered PBS (One-way ANOVA, n = 5/group, representative of 2 independent experiments). (**E**) The total number of BAL inflammatory cells was similar for hMSC-treated mice and infected control mice administered PBS (one-way ANOVA, n = 6/group). There was no significant difference in the total percentage of BAL (**F**) monocytes/macrophages and (**G**) neutrophils between mice administered hMCS and infected control mice administered PBS (one-way ANOVA, n = 6/group). Error bars represent interquartile range (IQR). ND = nondetectable.

To assess whether hMSC therapy decreased pulmonary inflammation, experimental mice were administered 2.5×10^5^ hMSCs or PBS via the tail vein, on day 2 or day 5 post-infection and sacrificed on day 7 post-infection. Uninfected mice were administered PBS intranasally. BAL fluid concentrations of IFN-γ, CXCL10, CCL2 and CCL5 were measured (Figure 5A–D). Cytokine/chemokine levels were below the limit of detection in BAL fluid of uninfected control mice. While influenza infection was associated with increased expression of all measured cytokine/chemokines, there were no significant differences between mice treated with hMSCs compared to control mice treated with PBS (Figure 5A–D). BAL inflammatory cell counts, including monocytes/macrophages and neutrophils, were also unaffected by hMSC administration (Figure 5E–G).

**Figure 6:**
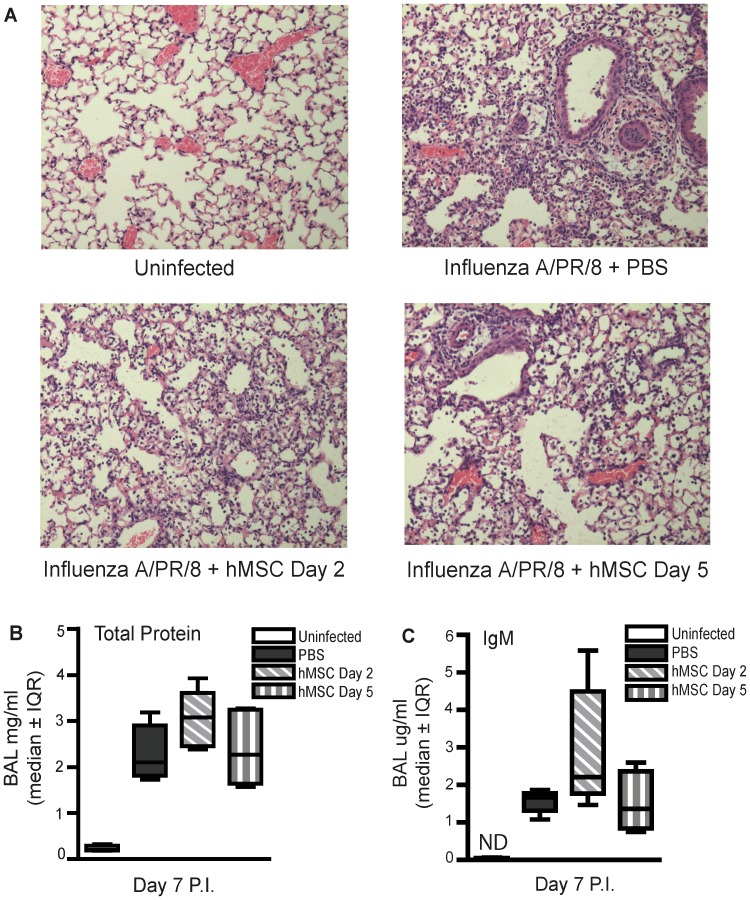
Therapeutic administration of hMSCs failed to modify pulmonary inflammation or alter acute lung injury in experimental severe influenza. Eight week old male C57Bl/6 mice were infected with 425 EID_50_ influenza A/PR/8 virus and administered 5×10^5^ hMSCs (passage 3) on day 2 or day 5 P.I. Mice were sacrificed and BAL was performed on day 7 P.I. (**A**) Representative images of hematoxylin and eosin stained lung sections demonstrate similar cellularity and lung injury in hMSC treated mice compared to infected controls. (**B,C**) No significant difference in markers of ALI (total protein or IgM) was observed between mice administered hMSCs on day 2 or day 5 P.I. and infected control mice administered PBS (one-way ANOVA, n = 5/group, representative of 2 independent experiments). Error bars represent interquartile range (IQR). ND = nondetectable.

On day 7 post-infection, similar inflammatory cell infitrates were observed in the lungs of hMSC treated mice compared to infected controls, as shown by histology (Figure 6A). Total protein and IgM were significantly lower or below the level of detection in BAL fluid of uninfected control mice compared to infected mice, respectively (Figure 6B,C). No difference in total protein or IgM was observed for mice administered hMSCs on day 2 or day 5 post-infection compared to control mice administered PBS (Figure 6B,C).

### Therapeutic Administration of hMSCs Failed to Alter Weight Loss or Improve Survival when used as an Adjunctive Therapy in Experimental Severe Influenza

To simulate the clinical presentation of influenza, combination antiviral therapy with oseltamivir and hMSCs was delayed for 48 hours after challenging C57Bl/6 mice with 425 EID_50_ influenza A/PR/8 virus. Mice were administered oseltamivir on days 2–6 post-infection, either alone or in combination with 5×10^5^ hMSCs administered on day 2 post-infection.

**Figure 7:**
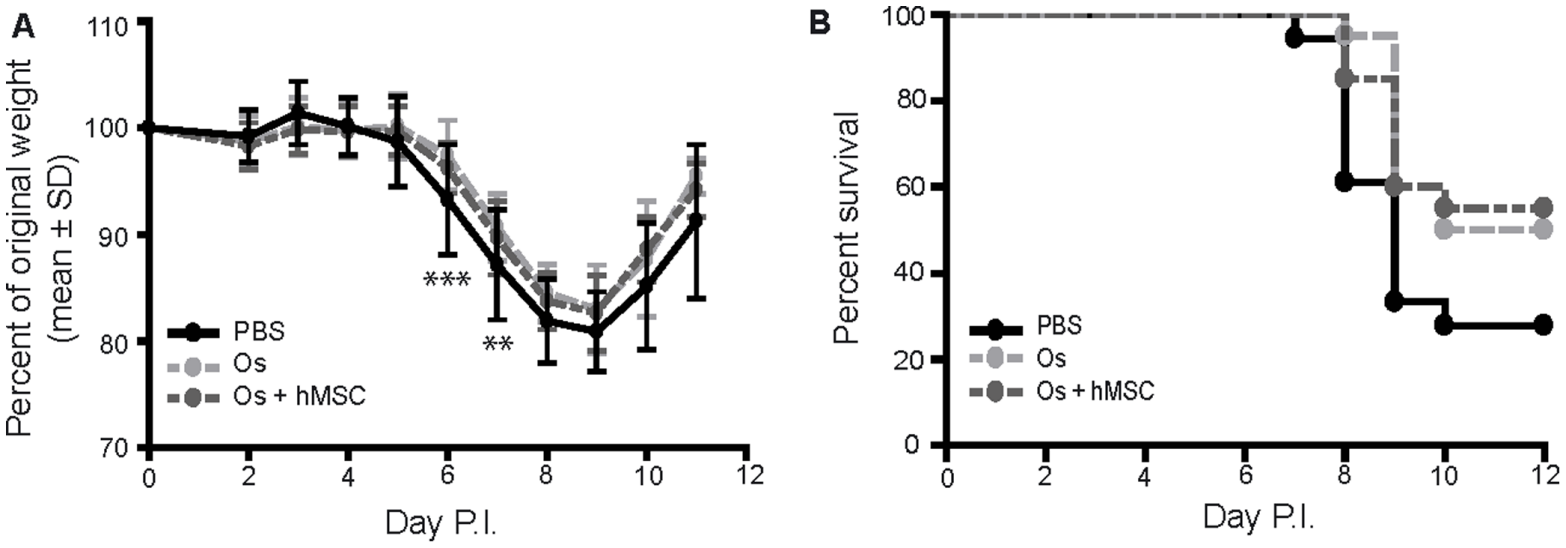
hMSC adjunctive therapy failed to alter weight loss or improve survival compared to antiviral therapy alone in experimental severe influenza. Eight-week old male C57Bl/6 mice infected with 425 EID_50_ influenza A/PR/8 virus were administered 2.5 mg/kg oseltamivir in 100 ul ddH2O via gavage, 1×daily for 5 days, beginning day 2 P.I., with or without hMSC administration (5×10^5^ cells) on day 2 P.I. (**A**) Weight loss kinetics over the course of infection were measured to assess morbidity in mice. Oseltamivir treated mice had significantly decreased weight loss compared to control mice on day 6 and day 7 P.I. (two-way ANOVA with Bonferroni post-tests, ***p<0.001, **p<0.01, n = 18–20/group; 2 pooled experiments). No difference in weight loss was observed between mice administered oseltamivir and hMSCs in combination compared to mice administered oseltamivir alone. Error bars represent standard deviation. (**B**) Survival curve. Mice administered oseltamivir were trending towards increased survival compared to the control group administered ddH_2_O; however, no difference was observed between mice administered oseltamivir and hMSCs in combination compared to mice administered oseltamivir alone (logrank test, n = 18–20/group; 2 pooled experiments). Os = oseltamivir.

On day 6 and day 7 post-infection, oseltamivir treated mice had significantly decreased weight loss compared to controls (Two-way ANOVA with Bonferroni post-tests, ***p<0.001, **p<0.01) (Figure 7A). No difference in weight loss was observed between infected mice administered oseltamivir and hMSCs in combination and infected mice administered oseltamivir alone. A trend towards increased survival of oseltamivir treated mice was observed (Figure 7B). No difference in survival was observed between infected mice administered oseltamivir and hMSCs in combination and infected mice administered oseltamivir alone (Figure 7B).

### Therapeutic Administration of hMSCs Failed to Decrease Pulmonary Inflammation or Prevent ALI when used as an Adjunctive Therapy in Experimental Severe Influenza

**Figure 8:**
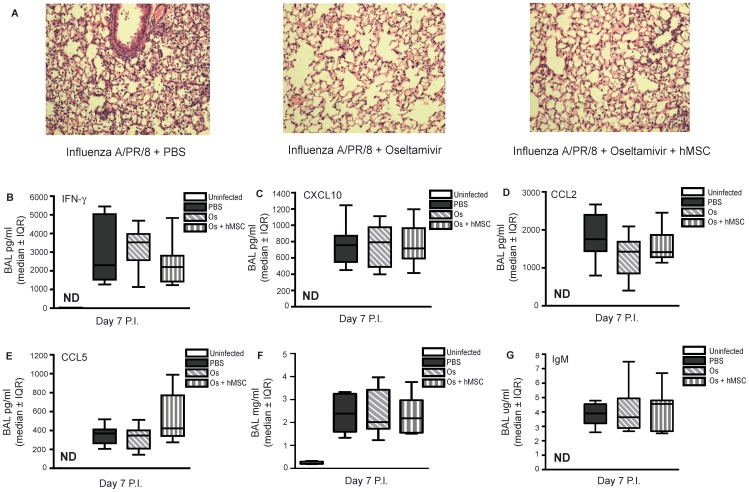
hMSC adjunctive therapy failed to decrease pulmonary inflammation or prevent ALI compared to antiviral therapy alone in experimental severe influenza. Eight-week old male C57Bl/6 mice infected with 425 EID_50_ influenza A/PR/8 virus were administered 2.5 mg/kg oseltamivir in 100 ul ddH2O via gavage, 1×daily for 5 days, beginning day 2 P.I., with or without hMSC administration (5×10^5^ cells) on day 2 P.I. and sacrificed on day 7 P.I. (**A**) Representative images of hematoxylin and eosin stained lung sections. Oseltamivir treated mice demonstrate decreased inflammatory cell infiltrates in the lung compared to untreated infected control mice. However, mice administered hMSCs and oseltamivir in combination demonstrate similar cellularity and lung injury to mice administered oseltamivir alone. (**B–E**) No significant difference in BAL fluid level of cytokines and chemokines (IFN-γ, CXCL10, CCL2 or CCL5) was observed for mice administered oseltamivir and hMSCs in combination, compared to mice administered oseltamivir alone (one-way ANOVA, n = 8–10/group, representative of 2 independent experiments). (**F,G**) No significant difference in markers of ALI (total protein or IgM) was observed for mice administered oseltamivir and hMSCs in combination, compared to mice administered oseltamivir alone (one-way ANOVA, n = 8–10/group, representative of 2 independent experiments). Error bars represent interquartile range (IQR). ND = nondetectable; Os = oseltamivir.

C57Bl/6 mice from each experimental group as described above were sacrificed on day 7 post-infection. Oseltamivir -treated mice demonstrate decreased inflammatory cell infiltrates in the lung as shown by histology, compared to untreated infected control mice (Figure 8A). However, inflammatory cell infiltrates and lung injury was similar between mice administered hMSCs and oseltamivir in combination compared to mice administered oseltamivir alone (Figure 8A). Compared with uninfected mice, we observed an increased level of all measured cytokine/chemokines in the BAL fluid of influenza A/PR/8-infected mice (Figure 8B–E). However, there were no significant differences in BAL fluid cytokine/chemokine levels between the infected mice treated with hMSCs and oseltamivir in combination and infected mice treated with oseltamivir alone. Similarly, there were no differences in BAL fluid total protein and IgM for infected mice treated with oseltamivir and hMSCs in combination compared to infected mice treated with oseltamivir alone (Figure 8F,G).

## Discussion

To our knowledge, this study is the first reported investigation of MSC therapy for treatment of influenza-induced ALI, specifically, or virus-induced ALI, in general. We observed that administration of mMSCs or hMSCs, either alone or as an adjunctive treatment strategy, failed to improve survival, decrease pulmonary inflammation or prevent ALI in experimental severe influenza. Despite similarities in the clinical presentation and pathobiology of ALI and severe influenza, our findings suggest that MSC therapy may not be an effective therapeutic strategy to improve outcomes in severe influenza.

The presence of local signaling molecules in the MSC microenvironment can play an important role in determining the efficacy of MSCs in pre-clinical models. Ren *et al.* demonstrated that under co-stimulation with IFN-γ, in the co-presence of either TNF, IL-1α or IL-1β, hMSCs secreted large amounts of indoleamine 2,3-dioxygenase (IDO) and chemokines, driving both T-cell migration and hMSC-mediated T-cell suppression [41]. In contrast, low levels of IFN-γ or the absence of certain signals in the MSC microenvironment can cause MSCs to behave as antigen presenting cells [45]. Because infections are characterized by distinct cytokine profiles and inflammatory micro-environments dynamically regulated throughout the course of infection, the timing at which MSCs were introduced to the host microenvironment in our model may have influenced MSC functionality. In our experiments, MSCs were delivered at various time points when lung inflammation was minimal or elevated, respectively. In all regimens (both prophylactic and therapeutic), MSCs failed to dampen host lung inflammation, decrease ALI, or improve morbidity and mortality. We also assessed mMSC efficacy after incubation with IFN-γ and TNF for 24 hours before harvesting for injection; however, this approach failed to improve survival in our murine model of severe influenza (data not shown). Taken together, these results indicate that although timing of MSC administration is likely important, it may not account for lack of MSC efficacy in our model of experimental severe influenza. Moreover, the cytokine milieu required for MSC immunosuppression *in vitro* may not sufficiently reflect the cytokine milieu required *in vivo*. For example, MSCs failed to improve outcome in three *in vivo* models of disease mediated by T-cell function, despite their ability to suppress T-cell proliferation *in vitro* [42]–[44].

Recent *in vivo* studies have shown that MSCs may enhance antimicrobial immune effector cell function and increase bacterial clearance during infectious challenge [28], [35], [46]. Therefore, MSC treatment could potentially provide clinical benefit in the setting of secondary bacterial pneumonia following influenza, an important cause of influenza-associated mortality, particularly in elderly patients [47]. Alternatively, it is possible that influenza virus interacted directly with MSCs to inhibit their anti-inflammatory activity. A recent study has shown that MSCs express influenza virus receptors α-2,3 and α-2,6 linked sialic acid, and support efficient infection and replication of influenza virus *in vitro* [48]. Influenza virus binding to MSCs resulted in MSC lysis and cytokine secretion. Interaction between the influenza virus and MSC-expressing TLRs may also be important. Temporary inactivation of MSC-mediated anti-inflammatory effects induced by viral activation of toll-like receptor 3 (TLR3) and TLR4 on MSCs has been described [49]. This observation may represent a mechanism that allows the immune system to function effectively in the presence of virus or bacteria. However, the influenza virus is comprised of single-stranded RNA (ssRNA) and does not generate significant amounts of double-stranded RNA [50] or LPS, the ligands for TLR3 and TLR4. Furthermore, TLRs important for ssRNA recognition (TLR7 and TLR8) [51], [52] were not expressed on bone marrow-derived human MSCs [53] but were expressed on bone marrow-derived murine MSCs [54].

Although MSC-secreted proteins such as TSG-6 and PGE_2_ have been attributed to MSC-mediated improved outcome in pre-clinical models of lung injury, these proteins may not be beneficial in the treatment of severe influenza, despite underlying pathophysiology common to lung injury derived from a viral or bacterial source. For example, although it may be beneficial to administer recombinant TSG-6 as treatment for LPS-induced lung injury in mice [38], TSG-6 treatment for severe influenza may be detrimental due to its ability to upregulate cyclooxygenase-2 (COX-2), as COX-2 upregulation has been associated with increased morbidity and mortality in severe influenza [3], [55], [56]. A similar rationale is applicable to MSC upregulation of PGE_2_, which is generated by conversion of arachidonic acid by COX-2.

Finally, the lack of therapeutic benefit of MSC therapy observed in this study could possibly be due to inherent limitations of the murine model of severe influenza. Specifically, the short duration of the murine severe influenza model does not allow for investigation of lung recovery following influenza infection. While administration of MSCs does not appear to affect clinical outcome in experimental severe influenza in the acute setting, the results of the current study do not preclude the possibility that MSC therapy could potentially contribute to long-term repair and restoration of full lung function following influenza infection.

### Conclusion

Despite accumulating evidence on the beneficial effects of MSC administration in pre-clinical models of ALI, this study indicates that MSCs may not be an effective therapeutic or prophylactic approach to decrease pulmonary inflammation, prevent ALI, and improve clinical outcome in acute severe influenza. As MSCs are currently entering clinical trials for treatment of sepsis and ARDS, this study provides a cautionary note that MSC treatment may not be applicable to all types of ALI. Specifically, the results of this study fail to support a potential role for MSC therapy in the prophylaxis or treatment of acute severe influenza. These findings have important implications for the design of clinical translational studies investigating MSC therapy for management of ARDS and pulmonary infections.

## Supporting Information

Figure S1 **MSCs fulfill ISCT defining criteria.** Phase contrast microscopy images of **(A, left to right)** undifferentiated mMSCs (P9), Oil Red stained mMSCs differentiated into adipocytes, Alizarin Red S stained mMSCs differentiated into osteocytes, and Alcian Blue stained mMSCs differentiated into chondrocytes (10×magnification). **(B, left to right)** Undifferentiated hMSCs (P3), Oil Red stained hMSCs differentiated into adipocytes, Alizarin Red stained hMSCs differentiated into osteocytes, and Alcian Blue stained hMSCs differentiated into chondrocytes (10×magnification). **(C)** Flow cytometry analysis of hMSC markers (P3). hMSCs (P3) were >99% positive for stem cell surface antigens CD73, CD90, and CD105 and <2% positive for hematopoietic cell markers CD11b, CD19, CD34, CD45, HLA-DR.
(TIF)
