# Supplementary figures and images for: Mesenchymal Stromal (Stem) Cell Therapy Fails to Improve Outcomes in Experimental Severe Influenza
Source: PLoS One. 2013 Aug 15;8(8):e71761. doi: 10.1371/journal.pone.0071761 (PMC3744455; doi:10.1371/journal.pone.0071761)

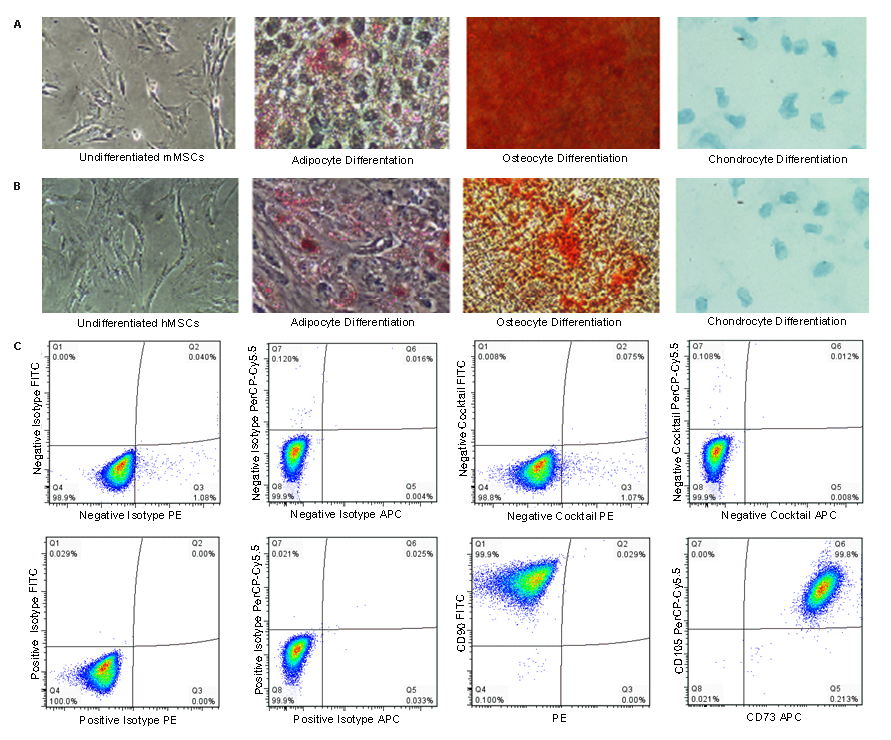

Supplement: Figure S1 — MSCs fulfill ISCT defining criteria. Phase contrast microscopy images of (A, left to right) undifferentiated mMSCs (P9), Oil Red stained mMSCs differentiated into adipocytes, Alizarin Red S stained mMSCs differentiated into osteocytes, and Alcian Blue stained mMSCs differentiated into chondrocytes (10×magnification). (B, left to right) Undifferentiated hMSCs (P3), Oil Red stained hMSCs differentiated into adipocytes, Alizarin Red stained hMSCs differentiated into osteocytes, and Alcian Blue stained hMSCs differentiated into chondrocytes (10×magnification). (C) Flow cytometry analysis of hMSC markers (P3). hMSCs (P3) were >99% positive for stem cell surface antigens CD73, CD90, and CD105 and <2% positive for hematopoietic cell markers CD11b, CD19, CD34, CD45, HLA-DR. (TIF) [file pone.0071761.s001.tif]
